# Kairomone and Camera Trapping New Zealand Flower Thrips, *Thrips obscuratus*

**DOI:** 10.3390/insects11090622

**Published:** 2020-09-11

**Authors:** David Maxwell Suckling, Mailee E. Stanbury, Ox Lennon, Kate M. Colhoun, Fabio Chinellato, Ashraf M. El-Sayed

**Affiliations:** 1 The New Zealand Institute for Plant & Food Research Limited, Private Bag 4704, Christchurch 8140, New Zealand; MStanbury@doc.govt.nz (M.E.S.); ox.lennon@vuw.ac.nz (O.L.); ashraf.el-sayed@plantandfood.co.nz (A.M.E.-S.); 2 School of Biological Sciences, University of Auckland, Auckland 1010, New Zealand; 3 The New Zealand Institute for Plant & Food Research Limited, 990 Earnscleugh Road, RD 1, Alexandra 9391, New Zealand; Kate.Colhoun@plantandfood.co.nz; 4 Department of Agronomy, Food, Natural Resources, Animals and Environment, University of Padova, Agripolis, Viale dell’Università 16, 35020 Legnaro (PD), Italy; chinellato.f@gmail.com

**Keywords:** thrips, phenology, lure, suppression, 6-pentyl-2H-pyran-2-one, 6-PAP

## Abstract

**Simple Summary:**

Camera traps using new insect attractant lures made from the smell of ripe peaches were used to provide daily counts of New Zealand Flower Thrips from online images of the sticky base of the traps. Software and manual counting were used to determine arriving thrips counts, which fluctuated daily but showed peaks in 2013 up to 1000 thrips in a trap in one day. Application of knockdown insecticides inhibited further thrips arrival in three peach blocks, according to the camera traps. Dose response experiments with the peach lure to attract thrips showed higher catches with more lure, within 24 h, up to 11-fold above the catch in unbaited traps. A 32-fold increase in thrips per tree was achieved over the control. Camera traps have huge potential in integrated pest management, by providing daily phenology without trap visits.

**Abstract:**

This project investigated how kairomone lures, camera traps, and counting software could together contribute to pest management. Images of cumulative daily catch of New Zealand Flower Thrips (NZFT) attracted to a ripe peach lactone (6-pentyl-2H-pyran-2-one; 6-PAP) were automatically loaded to the internet and compared with scanned bases checked weekly using in-house software and manual counting. Camera traps were able to provide thrips counts equivalent to delta traps, but daily and remotely. An 11-fold greater NZFT count occurred within 24 h in passive traps after polyethylene sachets loaded with 250 mg of 6-PAP were placed in trees. Intensive trapping, by placing 1, 2, 4, and 8 traps per tree (500 mg/trap), resulted in a maximum 32-fold increase in thrips per tree. While 6-PAP has proved to be a useful tool for monitoring NZFT numbers, our results suggest that it is not likely to be suitable for mass trapping. Future research should investigate NZFT behavior to better understand population movement on an area-wide basis. Camera traps can be a valuable tool for recording insect flight activity remotely, but the number of traps required for statistically reliable estimates may be prohibitive.

## 1. Introduction

Monitoring insect pests is normally necessary to determine the timing of insecticide treatment or other interventions by growers employing integrated pest management [1]. Such data are traditionally collected by manually checking and counting trap captures weekly, ideally with a lure or possibly colored traps [2,3]. Depending on how many traps are in a given system, this method requires labor and regular transport to each trap site. There is a risk of inconsistency between observers during insect counting and trap deployment, and significant time lags between counting and reporting. Remote devices including cameras for obtaining high quality images at set time intervals are increasingly used across different fields, including in vertebrate biology [4] and entomology, where automated insect identification has also been examined [5,6,7,8,9]. Remotely provided images avoid the need for people to go into the field to check traps, saving both time and travel costs. However, testing of prototype systems is needed to clarify how the technology could actually deliver benefits. Pests targeted with such systems based on lures include moths [10,11] with efforts commencing for other pests, such as weevils [7]. The potential uses clearly exist wherever lures and traps are used [12], although generic lures create additional challenges for species identification. We chose an abundant but tiny pest [13] with a powerful and selective new attractant [14] to develop our concepts of camera traps, using cameras re-deployed from the surveillance industry. The cameras were connected to novel in-house automatic counting software to deliver daily pest phenology.

Field tests of trapping, and especially mass trapping, have predominantly targeted pest groups other than thrips [15]. New Zealand flower thrips (NZFT), *Thrips obscuratus* [16] is an indigenous species and a pest of apricots, peaches, and nectarines at harvest. Thrips pose a problem through directly damaging fruit and flowers [16,17], and causing fruit drop or russeting. They also prove a vector to botrytis and brown rot [17]. NZFT can also be a market access pest [18,19] and may have impacts on grapes [20].

Thrips numbers on stonefruit in New Zealand have historically been controlled by use of applications of insecticides [21]. To remain competitive in a market where public awareness of the risks of insecticide use and residues on fruits is increasing, a novel means of managing pest insects is needed whilst eliminating or reducing insecticide treatment on fruit close to harvest. Obtaining more accurate and detailed data of population dynamics for a pest species is critical to the development of new methods of suppression.

6-Pentyl-2H-pyran-2-one (6-PAP) has been identified as a potent kairomone for NZFT [14]. It has been shown to double catch of NZFT in traps [22], compared with an earlier thrips lure, ethyl nicotinate [23]. Weekly trapping identified a single main flight peak in December–January [22]. We tested 6-PAP in a 4 × 4 array of one trap per peach orchard tree, but there was no evidence of trap competition at the spacing distance of one tree, despite catch of up to thousands of thrips per trap [24]. A previous weekly study of NZFT phenology from beating trays has suggested patterns of sudden build-up in the orchards [13]. Here, we had access to locally produced remote surveillance camera traps with internet uploading and storage of images, which we installed to investigate their performance and suitability in daily thrips phenology research. Specifically, we investigated the potential to detect a daily or weekly influx of thrips into the orchard, or a gradual build-up of the insects. Camera traps were also used to record the impact of knock-down insecticides. Our second objective, after investigating camera traps, was to test alternative ways of deploying 6-PAP. The dose response of NZFT to loadings of the 6-PAP kairomone was tested in single fruit trees, and the effect of trap competition was tested at high density (up to eight traps per tree), in order to detect upper limits of attraction.

## 2. Materials and Methods

### 2.1. Daily Thrips Phenology from Self-Reporting Camera Traps

**Figure 1:**
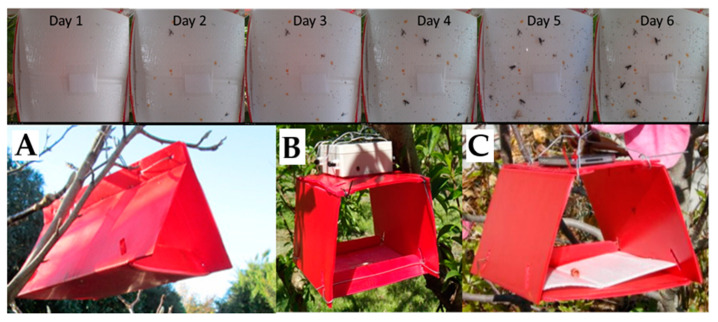
Camera traps tested with New Zealand flower thrips *Thrips obscuratus*, baited with peach lactone (6-pentyl-2H-pyran-2-one, 6-PAP) and used to upload cumulative daily catch photos (Top) for counting thrips by in-house software. (**A**) Standard Delta trap (T, each opening is 56 cm^2^), (**B**) C, Camera trap (213 cm^2^ openings with BioCamSR), (**C**) P, Smartphone-based prototype (244 cm^2^ openings to achieve focal length). The 6-PAP sachet lure for *T. obscuratus* is visible on the bases (top). Images were loaded online as in the related example from the prototype camera trap system and software application, Pest Spy, https://bit.ly/3id5pjU.

Trials from 7 November 2012 to 8 January 2013) and 30 November 2013–12 January 2014 were set out in a Southern Star peach block with a randomized block design with 10 m spacing between treatment traps, and 15 m spacing between replicate rows. Technical problems led to a later start in the second year but locations were consistent. Traps were hung in peach trees at 2 m above the ground in a research orchard in Clyde, Central Otago (−45.2024°, 169.3171°). Traps were based on traditional delta traps made up of a red plastic core flute body, each with an 18 × 19 cm sticky base inside [25] (Etec Crop Solutions Ltd., Auckland, New Zealand). Three types of trap were used (Figure 1, T, standard Delta trap; C, Delta trap with Camera; P, Prototype trap with smartphone). The sticky base area in each trap type was the same, but the area of each trap opening was 56 cm^2^ for the normal delta traps, 213 cm^2^ for the camera traps, 244 cm^2^ for the prototype traps. The slightly larger height of the prototype trap was to allow for focal length with future installation of a smartphone which uses a software application under development, “Pest Spy” (El-Sayed unpublished data). Each sticky base was baited with a 50 × 50 mm polyethylene bag containing a 40 × 20 mm felt strip onto which was dispensed 500 mg of the known kairomone 6-pentyl-2H-pyran-2-one (6-PAP) [14], obtained from Frinton Laboratories (Hainesport, NJ, USA), purity 96%. The lures were replaced fortnightly to ensure attractivity.

Nine traps (3 × 3 array) were placed out in the 2013–2014 season, consisting of six Delta traps (Figure 1) and three Camera traps (Figure 1B, traps with extra height to allow for appropriate focal length from bases). In the second year we used 20 traps (4 × 5 array) because this offered inclusion of more traps to help improve the power of the trial. Ten of these were Delta traps (Figure 1A); five were the Camera traps as used in the previous season (Figure 1B); five were Prototype (slightly taller) traps (Figure 1C). The layout was derived from an extended Latin square for four treatments, and at the time the trial was designed, it was expected that a camera would be associated with each C trap. However, only three cameras were available, so two traps did not have a camera.

Monitoring was conducted on a daily basis (Camera trap) and weekly basis (Delta and Prototype traps) from 8 November 2011 until 8 January 2012, and 1 December 2013 to 12 January 2014, to record data from the beginning of population growth through the seasonal peak [13]. Sticky bases of all traps were changed weekly, and NZFT individuals counted manually. Three BioCamSR cameras (Mi5 Security Ltd., Auckland, New Zealand) [26] were used, each taking a 3 megapixel image every 24 h and automatically sending them by General Packet Radio Services (GPRS) network to a dedicated web server. Pictures obtained daily by each camera trap were downloaded from the web page and NZFT individuals were manually counted on a computer monitor in full resolution where the number of thrips on a base was less than about 200–500. Where more thrips were estimated as caught on a base, in-house counting software using number of pixels and Red-Green-Blue color match estimated the number of thrips caught [27]. The counting software, which was found to be more accurate than manual counting above 200 thrips [27], will be reported in more detail elsewhere, but uses pixel count and RGB colour spectrum after training on samples. Count data from batch-processed standard images was output to an Excel file (El-Sayed unpublished). Lack of separation of morphologically similar insects can be a weakness but was resolved here by lure specificity.

Weekly catches totaled across 6 weeks were analyzed with a generalized linear model approach [28]. Two sets of analyses were carried out: first, the catches as recorded were analyzed, with a separate analysis for each week, and then an analysis of the totals per trap over the 6 weeks. The second analysis used the same seven sets, except that an adjustment for trap area was included. For both sets of data, some initial analyses (details not presented) were carried out to assess whether there was any strong spatial patterning (associated with rows of trees or position within row) in the data. The spatial factors were not found to be important, so were ignored in the final analysis.

In the final analyses, substantial over-dispersion [28] was found. Thus, the data were analyzed using a negative-binomial generalized linear model [28] with a logarithmic link. The aggregation parameter was estimated by fitting this model using a Poisson-gamma hierarchical generalized linear model [29], with individual trees (20 of these) as a random effect. For the analysis adjusting for trap area, an ‘offset’ of ln (area/56) was included in the model. An offset is a parameter-less explanatory variable [28], which allowed analysis of the actual trap counts, scaling the results to be in terms of counts equivalent to the area of a standard delta trap (56 cm^2^).

For both sets of analyses, the importance of fixed effects was assessed with a Χ^2^ test of the change in deviance on dropping a term, as implemented in GenStat’s HGFTEST procedure. Contrasts between the trap types were assessed similarly, but using HGFTEST. In the results, means are presented along with 95% confidence limits. These were obtained as predictions on the link (logarithmic) scale, and transformed to the count scale. For the area-adjusted analyses, the offset was set to 0 to give results in terms of the area of a standard delta trap (i.e., counts per 56 cm^2^).

### 2.2. Effect of Insecticides on Catch

We predicted that camera traps would record an immediate effect of 1-naphthyl-*N*-methylcarbamate (carbaryl) insecticide application due to its rapid action. A trial was conducted with three peach cultivars (cv Southern Ice, Coconut Ice, Southern Star) at the Clyde Research Centre (45.2031 S, 169.3170 E). The peach trees were at least 5 years old and close-planted, with 1667 peach trees/ha [13], and were within a 3.5 ha block of mixed white peaches. There were four rows of each cultivar, with cultivars alternating throughout the block. Sprays were applied by conventional air-blast sprayer, with Sevin Flo (500 g of carbaryl/L in suspension applied at 120 mL/100 L at a water rate of 700 L/Ha) (Etec Crop Solutions, Auckland, New Zealand). A spray over the whole block was applied on 2 February 2014. The first cultivar, Southern Ice was sprayed on 7 February and harvesting started on 9 February. The second cultivar, Coconut Ice was sprayed on 15 February and harvesting started on 18 February. Southern Star was sprayed on 22 February and harvested 26 February. One camera trap baited with 500 mg 6-PAP as above was installed in one row for each cultivar from 29 January 2014 to 5 March 2014, and thrips numbers counted daily from images as above.

### 2.3. Dose Response of Thrips to Different 6-PAP Kairomone Loadings in Trees

Unbaited sticky traps made from clear transparency film (29 × 21 cm) were covered on one side with clear sticky glue. These were hung vertically in the middle of each tree at about 1.5 m above the ground for 4 days (1–5 February 2013). The traps were then brought into the laboratory, and thrips catch per trap was counted manually. Treatments were then applied to cv Clutha Gold apricot trees on 5 February 2013. Treatments consisted of 6-PAP applied to felt squares encased in polyethylene bags [22], in doses of 0, 50, 100, 150, 200, 250 mg of 6-PAP/tree. Lures containing the different concentrations were hung in the middle of each tree, approximately 1 m above the position where the first sticky trap had been. Twenty-four hours after the application of the lures, a second set of vertical panel sticky traps were hung in the trees. These were hung in the middle of each tree (in the same position as each of the first traps) for 4 days after the treatments were applied, then were brought into the laboratory and the thrips on each trap counted manually (6–10 February 2013). Five replicates were deployed within one block of the commercial orchard using a randomized block design. Harvesting of some fruit took place while the trial was underway. The first pick was completed by the start of the trial, the second and third picks took place during the trial, and the fourth pick took place after the trial finished. Thus, some fruit were left on the trees for the duration of the trial.

Data for the pre- and post-treatment traps were analyzed within a single analysis. The approach was similar to that used for the camera trapping trial: some initial analyses were carried out to assess the importance of any spatial trends relating to replicates, tree row, or tree position within the row. However, these spatial factors were not found to be important (details not presented), so were ignored in the final analysis. In the final analysis, since there was substantial over-dispersion (potentially relating to thrips not behaving independently), the data were analyzed using a negative-binomial generalized linear model (GLM) with the aggregation parameter estimated by fitting this model using a Poisson-gamma Hierarchical GLM (HGLM), with individual trap (60 of these) as a random effect. To account for the pairing of traps (one pre- and one post-treatment trap per tree), Tree was included as a random effect.

Initially, the six kairomone rates were included as a factor, as a fixed effect. To explore the relationship between count and rate, rate was then included as a linear contrast. Since the linear contrast is fitted on the logarithmic scale, this is essentially fitting an exponential relationship to rate. The contrast was fitted to the data post-lure only, and an approximate R^2^ for this contrast was calculated as the square of the correlation between the dose means and the fitted values for each dose. All analyses were carried out with GenStat [30].

### 2.4. Effect of Trap Density in One Tree on Catch

This trial was carried out in an insecticide-free mixed peach/nectarine orchard after harvest at Little River on Banks Peninsula (43.7726 S, 172.7890 E), to assess the effect of increasing numbers of traps per tree, and thus decreasing distance between traps within a tree, aiming to assess whether there is any inhibitory effect of nearby traps on catch. Treatments varied the number of traps per tree, each with the same 500 mg kairomone lure on a sticky base [22] (1, 2, 4, or 8, with about 80, 56, and 40 cm between traps for treatments 2–8), with 10 replicates for each treatment. There were thus 40 trees used in total, which were arranged in a 4 × 10 grid using a randomized block design. There were two trees between treated trees in both directions, equivalent to a 12 m spacing between trees within a tree row, and 15 m between replicate rows. Sticky bases were checked and replaced after 2 weeks and then collected after a further 2 weeks (4 weeks). Thrips numbers were counted from images as above. The total catch (2 weeks + 4 weeks) was calculated for each trap. Numbers at each of the two assessments, and also the total over the two assessments, were analyzed separately. Some initial analyses were carried out to assess the importance of any spatial trends relating to replicates, or to cardinal trap position (W, NW, N, NE, E, SE, S, SW) within a tree. However, these spatial factors were not found to be important (details not presented), so were ignored in the final analysis. Since there was substantial over-dispersion (probably relating to thrips not behaving independently), the data were analyzed using a negative-binomial generalized linear model with the aggregation parameter estimated by fitting this model using a Poisson-gamma HGLM, with individual trap (150 of these) as a random effect. To account for the grouping of traps (1–8 traps per tree), Tree was included as a random effect. No attempt was made to model the change in catch per trap with increasing numbers of traps per tree: with only four different numbers of trap, such a model would need to be very simple (probably linear), and therefore not useful.

## 3. Results

### 3.1. Daily Thrips Phenology from Self-Reporting Camera Traps

**Figure 2:**
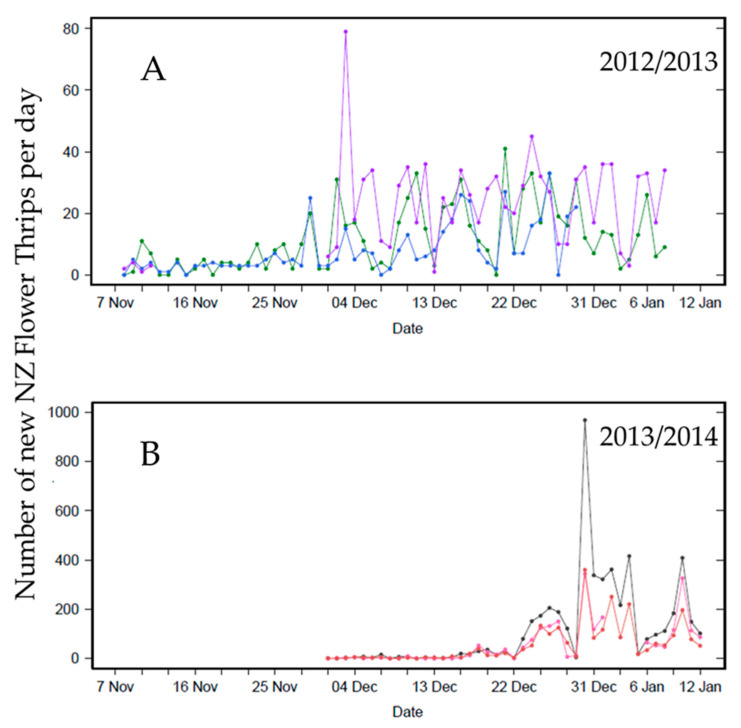
Number of New Zealand flower thrips (NZFT) caught each day over the peak population growth period for two seasons (**A**) 2012/2013 and (**B**) 2013/2014 in three self-reporting camera traps in a Central Otago, New Zealand stone-fruit orchard. Each color represents a trap.

The phenology patterns of NZFT in Central Otago were different in the two years of the study (Figure 2). The timing of the first peak differed between seasons, the major population peak occurred around 3 December in 2012, but did not occur until about 27 days later around 30 December in 2014. The numbers of NZFT caught in each season also differed (note difference in scales, maximum 80 and 1000), the largest catch in a single trap in one day was 78 thrips in 2012, and 950 in 2014. The mean daily catch per camera trap (SD) was 12 (10) thrips per trap per day in 2012–2013 and 77 (120) in 2013–2014.

**Figure 3:**
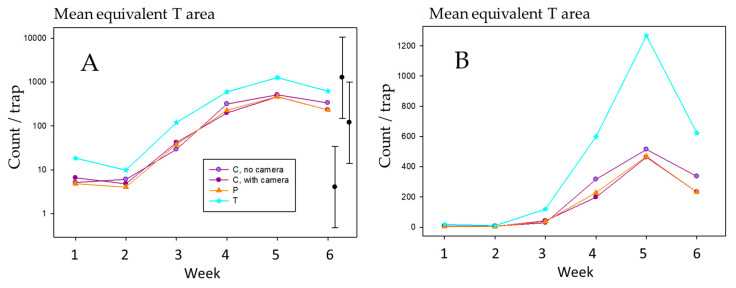
Corrected mean weekly count of *Thrips obscuratus* by trap type per standard 56 cm^2^ Delta trap (T) opening from 8 December 2013–12 January 2014 at Clyde Research Orchard. (**A**) Log scale. Error bars show confidence limits for the smallest, largest, and a mid-range mean within the plot. (**B**) *x*-axis on normal scale. Confidence limits are omitted on the count scale, as the highest limit is very large relative to the means, and the patterns amongst means are therefore obscured. T is the Delta trap, P is prototype, and C is the camera trap.

When adjusted for trap opening area, differences between the trap types were apparent (*p* = 0.004, 0.199 for weeks 1 and 2; *p* < 0.001 for the remaining weeks in 2014, for an overall test for differences between the four trap types); significance was entirely due to the higher proportional catches in the Delta traps (Figure 3) linked to the smaller entry (Figure 1). At no time were the counts for traps with cameras substantially different from those for traps without cameras (*p* = 0.843, 0.916, 0.522, 0.234, 1.000, 0.139 for the 6 weeks, respectively).

### 3.2. Effect of Insecticide Applications on Thrips Catch

**Figure 4:**
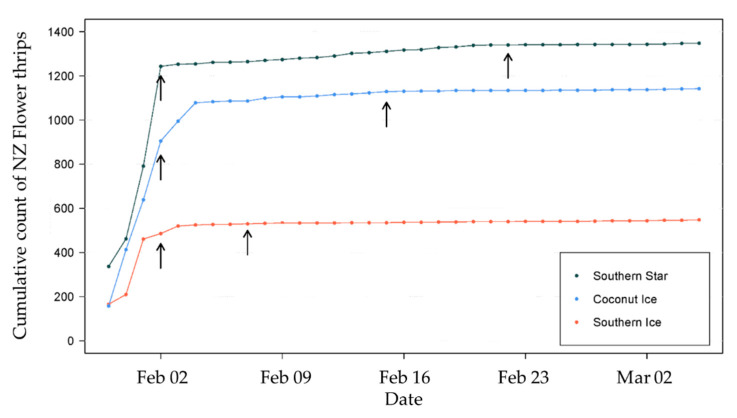
Effect of insecticides in three blocks with different varieties of peaches, on daily cumulative catch of *Thrips obscuratus* monitored by camera traps. Arrows indicate insecticide applications, which were timed to reduce incidence before harvest commenced.

Three camera traps reported rising levels of thrips in the three peach varieties at the end of January, but the application of carbaryl insecticide had a similar effect on catches in each block (Figure 4), with no increase in catch from 1–2 days after spray for an extended period. The additional insecticides appear to have had little additional benefit except for the Southern Star block, where the numbers were higher and had continued to rise very slowly after the first application, approaching harvest.

### 3.3. Dose Response of Thrips to Different Kairomone Loadings in Trees

**Figure 5:**
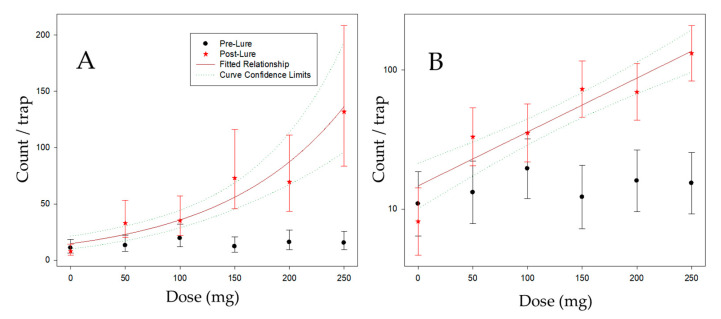
Mean counts of *Thrips obscuratus* per trap for each dose, pre- and post-lure, with fitted dose-response curve for the post-lure counts recorded after 24 h post lure placement. Error bars/dotted lines are 95% confidence limits for the means/fitted curve. (**A**) On the natural (count scale); (**B**) *Y*-axis log scaled.

The number of thrips caught in each tree by passive traps increased after adding the attractant to the middle of the tree in each positive treatment, compared to the initial passive trap catch (Figure 5). The number of thrips that were caught on traps within each tree increased in direct proportion to the amount of attractant placed in the middle of each tree, up to an 11-fold increase and following a log dose response (Figure 5).

There were no strong patterns associated with tree rows, tree positions, or replicates. As would be expected (Figure 5), there was a large interaction (*p* < 0.001) between Time (Pre/Post) and Dose (as a 6-level factor), indicating that the dose-response varied between the Pre and Post lure positioning assessments. Pre lure positioning, trap catch did not vary with dose (*p* = 0.628), but Post lure positioning, catch varied substantially between the doses (*p* < 0.001). A trend fitted to the doses for the Post catches (linear on the log Count scale) explained most of the variation in catch (R^2^ = 89%). However, there was significant lack of fit (*p* = 0.029) between this curve and the dose means, indicating that there was substantial deviation of some means from the linear trend. Primarily, this lack of fit is related to the two pairs of doses (50, 100; 150, 200) that had similar mean counts within the pair. It is also related to the relatively low catch of traps with no kairomone (dose = 0). The fitted curve (parameter (s.e.)) is log_10_ (Count) = 1.167 (0.079) + 0.00387 (0.00050) × Dose, which is equivalent to Count = 14.69 × 1.00895 ^Dose^.

### 3.4. Effect of Trap Density on Thrips Catch

The total number of thrips caught in each tree increased as the number of traps with attractant increased. The mean total caught per tree is 433, 1657, 8255, and 13,815 for 1, 2, 4, or 8 traps per tree (1, 1.9, 4.8, and 4 fold more per trap as trap density per tree increased from one to eight traps per tree (Total Counts × 1, 2, 4, or 8 traps per tree). Numbers on traps for density = 8 were 88%, 74%, and 84% of those on traps with density = 4 for 2 and 4 weeks and the total, respectively. Numbers per trap varied with traps per tree (‘density’) at both 2 and 4 weeks (*p* = 0.003, *p* = 0.001 for an overall test, respectively), and thus also for the overall total (*p* = 0.003). Therefore, even though there were fewer caught per trap, trees with eight traps per tree would catch around 7-fold as many thrips as trees with four traps, and almost 32-fold as many as were caught with a single trap per tree.

Numbers per trap increased from density = 1 to density = 4, but then reduced slightly between four and eight traps: however, this decrease was not significant (*p* = 0.560, 0.242, and 0.611 for 2 and 4 weeks and the total, respectively). If there was no interaction (negative or positive) between traps in a tree, it would be expected that there would be similar numbers caught per trap, regardless of trap density. Clearly, there is a positive effect on catch per trap with increasing numbers of traps, but the degree of positive effect was attenuated for a tree with above four traps (since there were no arrays of 5, 6, or 7 traps, it is not possible to identify where the effect occurs).

## 4. Discussion

Here we combined three new technologies, novel lures, camera traps, and counting software, to investigate their potential for improving pest management outcomes on a thrips pest of tree fruits, which is a notably challenging target due to a very wide range of floral hosts in the wider landscape ecosystem and a high level of mobility of this insect. In the orchard, the thrips are reported by growers as highly mobile in tracking the ripening fruit, and fruit harvest likely has impacts on thrips density which were not observed directly, although fruit were present during all the trials. Differences in thrips density between camera traps present within three different peach varieties could be due to many factors which would require more extensive study, including tree to tree or varietal differences in populations.

Attraction to rising emissions of 6-PAP and probably other odor signals is used by thrips to find ripe fruit. Another natural product attractant identified for this flower thrips species is cis-jasmone, for example [31]. Given their diverse host range [17], thrips must respond to many such volatile cues. A gradual build-up of population density occurred in the orchard in early summer in both years, although in the second year, peaks were evident for the first time. There was some evidence of three potential generational peaks in the second year, but this could be confounded by changes in arrival rate and movement from different alternative hosts in the landscape.

Daily phenology and density information is potentially very valuable to aid the interpretation of the process of plant or crop colonization. Davison and Birch [32] destructively sampled roses daily for nearly 5 years in Adelaide for *Thrips imaginis* and were able to determine that the numbers of thrips in roses varied from day to day in relation to (a) the natural growth of the population; (b) the influence of the weather throughout the year on the growth of the population; (c) the influence of the weather at about the time that the sample was taken on the activity of the adults in seeking out flowers. Such labor-intensive work is less frequently reported but camera traps might be tool that can offer a palatable substitute. Initially, direct crop sampling for some pests may have advantages for setting action thresholds, as in onion thrips *Thrips tabaci* [33], but with the increasing development of lures and traps this could change.

Camera traps coupled with software or manual counting were successful at detecting and quantifying the daily arrival of thrips, but these prototype units had major limitations including some battery life performance issues and high cost (>$1000), which will limit their specific adoption from the security industry supplier. Development of Pest Spy, which is an application based on using certain recycled cell phones for their communication, weather sensor and image transfer capability, represents one way forward with images taken every minute, or daily if desired (details from AM El-Sayed). In future, traps based on wing beat frequency may offer advantages also.

If critical pest events, such as an upsurge of numbers in the crop, could be detected in this way, a daily rather than weekly response would be possible by growers and orchardists, avoiding the greater losses likely if the pest is in the crop for longer. Likewise, knowledge that an intervention worked is valuable also, whether it is with an insecticide (demonstrated in this case), or other applications such as a sudden termination in moth catch in sex pheromone traps from the application of mating disruption [34], in that system, which can now tackle up to four pest species from a single device [35,36]. Knowledge of any moth flight during the disruption treatment would be of immediate interest to growers. The development of this technology is likely to reduce trap servicing costs, although alternative lower power and bandwidth systems that are not based on image processing may be more cost-effective. The development and implementation of economic thresholds and economic injury levels represent a major opportunity to improve immediacy and reduce response time from events recorded by camera traps for thrips and other insects. Issues such as trap saturation from insect catch will also require solutions to increase automation.

Mass trapping is a challenging technology for pest suppression in the face of immigration in the open field [15], but new socially acceptable tactics are needed for pest suppression in orchards, in order to meet rising expectations of consumers of horticultural commodities. While entomologists need objective reasons for alternatives to insecticides such as resistance, impact on non-target insects, and reduced environmental and human exposure to toxins, many consumers of fresh fruit are not unaware of these risks. Thrips have seldom been investigated compared to Lepidoptera and Coleoptera, for example, when it comes to odor-based monitoring or especially control in the open field [12,37]. Mass trapping with colored panels alone has been investigated in closed systems [38], perhaps rather optimistically. Some thrips researchers have suggested that small (2–3) fold increases over catch in colored panels by the addition of lures are useful [39], but any evidence of population suppression or prevention of damage is lacking [40], so it hard to understand the optimism even there. Weak attraction seems unlikely to enable the thrips lure proponents to advance the goal of mass trapping, even for closed populations [39]. Mass trapping closed or glasshouse thrips populations might have better prospects with aggregation pheromones [41], but reports of effective pest suppression from mass trapping thrips with other lures cannot be corroborated [38,42].

Here, the improvement by 6-PAP over unbaited lures was 11-fold at the highest loading. The open field problem in the Central Otago orchard environment is immigration of potentially extraordinarily large numbers of insects coming from other plants and highly attracted to fruit odors. The range of the 6-PAP lures appears to be relatively close, given the absence of competition at the tree to tree spacing, with only limited competition appearing at eight traps per tree. This increase in number of thrips per trap and per tree with more attractant does not support the use of mass trapping (although this has not been assessed directly), since the numbers apparently remained overwhelming in the environment. The maximum number of new thrips arriving in a single day was close to 1000 in one camera trap, which is remarkable at a certain level. In a practical sense, the traps were also able to show the impact of broadcast insecticides in the orchard by preventing any further thrips catches thereafter for several weeks.

There was clearly extensive local movement of thrips in the orchard, and clear evidence of odor-mediated movement, from the rise in catch in unbaited blank traps after the presentation of the lures nearby, as a function of dose. The use of 6-PAP reported by El-Sayed et al. [14] and elaborated by Allen et al. [22] was here validated as an excellent lure for this species, but the sheer number of thrips is clearly overwhelming to suppression using it. These result are consistent with a trapping experiment in a 4 × 4 trapping array at one tree spacing, where no difference in catch of thrips to 6-PAP was observed between 16 traps, unlike results from three other insect orders with sex pheromones, where a high corner-to-center ratio indicated trap competition essential for mass trapping [24]. The use of 16-trap arrays was proposed as a way of screening lures for suitability for further development of mass trapping or not, but the thrips-kairomone system showed no trap completion at one tree distance. This very short distance of the behavioral attraction effect was confirmed in unsuccessful attempts to pull thrips between adjacent trees in the orchard. Here we placed up to eight lures and traps in single trees, to determine whether the catch would continue to increase. Whilst we found a drop in the rate of increase in catch per trap, the numbers per tree caught continued to rise with more attractant present. This could seemingly support the development of a lure and kill approach but in the open field would require very large amounts of attractants (the top treatment used 4 g per tree of the 6-PAP). The short range of the lure and yet its powerful effects as an attractant are currently paradoxical, and thrips biology requires more research to unravel.

## 5. Conclusions

Despite high catches in our traps and removal of up to 140,000 thrips per tree, there was no evidence in support of the potential for mass trapping effects that could diminish orchard populations. Other applications for control using this kairomone remained to be explored, but clearly more release of the odorant brings more of the very highly mobile thrips. Wider availability of more cost-effective camera or other sensor-based trap systems would enable more pest management applications to approach real-time decision support for growers and increase knowledge of pest biology. For camera traps to truly impact upon IPM they need to offer some form of decision support to warrant the cost. Specific applications and use patterns will need to be identified, in the form of decision thresholds for growers in response to the new information, as well as the demonstration of the value of any intervention. As the technology evolves, reasons to service the trap should be logically removed as far as possible, by the development long life lures and non-saturating systems, as well as low power consumption.
